# Domestic Medicine

**Published:** 1871-07

**Authors:** 


### USEFUL RECIPES FOR THE HOUSEHOLD.

HOW TO REMOVE DANDRUFF.—Go to your druggist and have him prepare you the following formula:

Take—
Acid carbolic, half drachm;
Oil of Bergamot, one drachm;
Glycerine, two ounces.

Mix, and rub thoroughly into the roots of the hair; wash it out with soap and water, then apply bay rum freely. Use it once a week, it will keep the scalp clean, and eventually prevent the excessive formation of dandruff.

---

HOW TO MAKE BAY RUM.—Bay rum is a useful, agreeable and expensive application to the scalp. Everybody should use it though, so we will give a formula for making it—as good as can be purchased anywhere, and at a small cost:

Take—
Oil of Bay, 10 fluid drachms;
Oil of pimento, 1 fluid drachm;
Acetic ether, 2 fluid ounces;
Alcohol, 3 gallons;
Water, 2½ "

Mix, and in two weeks filter it carefully, when you will have a superior article of bay rum, better than can be purchased at an extravagant price, already prepared.

---

FLORIDA WATER is made in the following manner:

Oil of bergamot, 3 ounces;
Oil of cinnamon, 4 drachms;
Tincture of benzoin, 2 ounces;
Alcohol, 30 per cent. Baume, 1 gallon.

Mix and filter. In this manner you can secure an excellent article of Bay Rum or Florida Water, at a cost that will enable you to use it freely.

---

TO REMOVE FRECKLES AND TAN.—The following will be found a useful and harmless application to the face for whitening and beautifying the skin:

Take—
Fine pale honey, 4 ounces;
Glycerine, 1 ounce;

Mix them thoroughly by gentle heat, and when cold add—

Alcohol, 1 ounce;
Essence of ambergris, 6 drops;
Citric acid, 3 drachms.

Place the mixture in a pomatum dish, when it will be ready for use. Apply a small quantity to the face every night.

---

HABITUAL COSTIVENESS.—Ask your druggist to prepare the following pills. Take one every night before retiring. They will not act as a cathartic, but will gradually restore the bowels to their natural condition. They are particularly applicable to the constipation of women:

Take—
Extract of aloes, half-drachm;
Extract of nux vomica, 6 grains;
Extract of hyosciamus, 1 scruple;
Pulv. ipicac, 40 grains.

Mix, and make into twenty pills.

---

CONSUMPTION.—In the early stage of consumption, the following will be found to be beneficial in allaying the cough, and will often arrest the disease; it is harmless, and is not unpleasant to take:

Take—
Glycerine, 2 ounces;
Iodide of potassium, 1 drachm;
Morphine, 2 grains.

Mix, and take a teaspoonful before each meal, and at bedtime.

In the more advanced stage of the disease, the following will be found more efficacious:

Take—
Glycerine, 2 ounces;
Syrup of Iodide of Iron, half an ounce.
Morphine, 2 grains.

Mix, and take a teaspoonful every four or six hours.

---

DIARRHŒA IN CHILDREN.—Now that hot weather is upon us, our children will be complaining of bowel difficulties, the result of too free indulgence in berries and unripe fruit, or too violent play in the heat of the day. It is proper that your children should be allowed to run out of doors freely, but some shady place should be provided for them, else should they be kept within doors during the middle of the day, where they may not be prostrated by the excessive heat of the sun. Much of the sickness of children is produced by carelessness in allowing them to play freely in the heat of the sun, in the middle of the day.

If your little one complains of pain in the bowels, with a disposition to diarrhœa, strip him, put him to bed, place a towel, wet in cold water, to his head, and give him a few drops of the following mixture, which should always be kept in the house during the hot season:

Take—
Tincture of capsicum,
Tincture of camphor,
Tincture of rheubarb, of each, 1 ounce,
Tincture of opium, half an ounce.

Mix, and give a child four years old, six drops on a lump of sugar, every two hours, until pain is arrested.

The same medicine, in larger doses, will be found useful and timely in the pains and diarrhœas of older people.

---

HOW TO MAKE A WEATHER GLASS.—Go to your druggist and have him supply you with a thin glass tube, twelve inches long, and three-fourths of an inch in diameter,—such as are used for barometers. Have him make you the following solution:

Camphor, 2 drachms;

Nitre, 1½ "

Sal Ammoniac, 1 drachm;

Proof spirit, 2¼ ounces.

Allow this to dissolve, then fill your tube three-quarters full. Cork and seal it tightly, when it will be ready to indicate the state of the weather equal to any of the best barometers. As a sign of *fine* weather, the sediment of white flakes will settle near the bottom of the tube, while the liquid will be quite transparent above. As a sign of *rain*, these flakes will rise to the surface of the solution. At the approach of a *storm*, the matter will float on the surface in the form of white flakes, and the fluid will appear in a state of fermentation. During *frost*, the solution will present a starry appearance, and during the *summer* or *hot weather*, the matter will fall to the bottom as a solid substance.

So here you have a cheap and reliable barometer. Try it.

---

A PLEASANT REMEDY FOR SEA-SICKNESS.—There have been many suggestions made as to the prevention of sea-sickness, none of which have, to say the least, been found completely successful in practice. The introduction into practice of hydrate of chloral, which produces with certainty sleep for a definite number of hours, has suggested a means of escaping the horrors of a short sea-passage at least, and possibly of mitigating the most prolonged horrors of sea-sickness. An ordinary dose of hydrate of chloral produces sleep usually in a quarter of an hour, and with almost unfailing certainty. Some cases just published, by Dr. Doring, of Vienna, seem to show that the value of hydrate of chloral to obviate sea-sickness is very great. It produces quiet and prolonged sleep. In all the instances recorded, it seems to have been of great value, even during prolonged sea-voyages, giving a good night's rest, arresting violent sickness when it had set in, and stopping the tendency to its recurrence.

Don't take it, though, without the advice and consent of your physician, who will also inform you of the proper dose to take.

---

A BAD BREATH.—The popular term "bad breath," is a very significant expression for this unpleasant condition. What is more offensive to the acute olfactory sense than a fetid breath? It engenders a feeling of aversion and disgust, which is not readily overcome.

Great care should be exercised in keeping the mouth free from all extraneous substances. After each meal, a quill or ivory tooth-pick should be used, to remove any aliment that may have become lodged in the teeth during the process of mastication, and the mouth rinsed with tepid water. Every night previous to retiring, the teeth should be cleansed with a soft tooth-brush and water. As a rule, tooth pastes and powders should be eschewed as harmful agents.—If a dentifrice is desired, a little fine toilet soap, or charcoal reduced to an imppalable powder, may be used. This is all that will be required. Decayed teeth are a very prolific source of bad breath. As soon as it is ascertained that a tooth is affected, it should have immediate attention from some competent dentist.

Carious teeth are often the source of serious functional and general disturbance. It sometimes occurs that persons with a number of defective teeth are constantly ailing with either gastric or nervous troubles, when, upon a removal of these unsound members, all the unpleasant symptoms promptly disappear.

It may be well to give a word of caution in regard to diet; by irregularities in eating, the digestive functions become greatly impaired, and for want of proper digestion, the aliment undergoes zymotic change, during which process noxious gases are evolved, and cause a foul breath. When cases arise from disease, it is either of the stomach, lungs, or the respiratory passages. In these cases a physician should be consulted at once.

Many substances are in vogue to sweeten the breath, and to disguise any unpleasant scent, as of spirits, tobacco, etc. With the vulgar it is customary to use some pungent aromatic, as cloves, etc., but this savors too strongly of the drinking-bar to be used by any but tipplers.

